# Rare presentation of Rosai–Dorfman disease with severe lymphopenia: a pediatric case report and review of the literature

**DOI:** 10.1007/s00432-026-06496-8

**Published:** 2026-05-11

**Authors:** Doa’a Abdalla, Ruth Radcliffe, Hasan Asfour

**Affiliations:** 1 Department of Paediatric Oncology, Leicester Royal Infirmary, University Hospitals of Leicester NHS Trust, Leicester, UK; 2 Department of Immunology, Leicester Royal Infirmary, University Hospitals of Leicester, Leicester, UK; 3 Leicester Cancer Research Centre, College of Life Sciences, University of Leicester, Clinical Sciences BuildingLeicester Royal Infirmary, Leicester, LE1 5WW UK

**Keywords:** RDD, Lymphadenopathy, Cytopenia, Histiocytosis, Pediatric oncology, Immune dysregulation

## Abstract

Rosai–Dorfman disease (RDD) is a rare type of histiocytosis usually presenting with painless bilateral cervical lymphadenopathy and leukocytosis. Cytopenias, particularly isolated lymphopenia, are particularly uncommon and poorly characterized. Hereby, we report a case of a 3-year-old boy presenting with progressive bilateral cervical lymphadenopathy and profound T- and B-cell lymphopenia. Immunophenotyping demonstrated markedly reduced CD3^+^, CD4^+^, CD8^+^, and CD19^+^ cells, low borderline level of NK-cells, and expansion of TCRγδ^+^ double-negative (CD3^+^/CD4^−^/CD8^−^) T cells. B-cell subset analysis revealed reduced switched memory and marginal zone compartments with increased transitional B cells, suggesting immune dysregulation. Imaging confirmed extensive cervical and mediastinal lymphadenopathy. Lymph node excision biopsy showed characteristic sinus histiocytosis with emperipolesis, confirming the diagnosis of nodal RDD. No autoantibodies were detected, and bone marrow examination and genetic testing were unremarkable. Given the clinical stability and absence of organ dysfunction, the patient was conservatively managed with prophylactic antimicrobials and close surveillance, remaining stable at follow-up. A review of the literature was also conducted, identifying eight well-characterized RDD cases associated with cytopenia, involving only two cases reporting isolated lymphopenia, emphasizing the rarity of this presentation. Most cases required systemic immunosuppression due to autoimmune features or progressive disease. Detailed lymphocyte subset characterization was rarely reported. In conclusion, this case expands the immunologic spectrum of RDD and highlights isolated severe lymphopenia as a uniquely rare presentation. Comprehensive immunophenotyping is essential to distinguish RDD from primary immunodeficiency and lymphoproliferative disorders, as immune-dysregulated RDD may represent a biologically distinct subgroup with implications for precision management.

## Introduction

Rosai–Dorfman disease (RDD) is a rare condition that was first described in 1969 as sinus histiocytosis with massive lymphadenopathy (Doglioni 2021). Histologically, RDD is characterized by the abundance of S100^+^, CD68^+^, CD1a^−^, and CD207^−^ histiocytes, indicating the hallmark of emperipolesis, where intact cells like neutrophils and lymphocytes are engulfed by histiocytes (Bruce-Brand et al. 2020). This disease is currently classified as “histiocytoses of the R group”, encompassing familial, nodal, extranodal, neoplasia-associated, and immune-disease-associated subtypes (Emile et al. 2016).

The RDD mainly affects children and young adults with male predominance. This disease typically presents with painless bilateral cervical lymphadenopathy, often associated with fever, leukocytosis, elevated erythrocyte sedimentation rate, and hypergammaglobulinemia (Abla et al. 2018). Extranodal involvement occurs in up to 40% of cases and may affect skin, oral cavity, nasal cavity, eyes, bone, central nervous system, genitourinary system, and respiratory tract (Deen et al. 2022).

The etiology of RDD remains unclear. Despite no clear evidence, multiple reports associate RDD with viral infections, including Epstein-Barr virus, herpes viruses, HIV, and COVID-19 (Khan et al. 2003; Sall et al. 2015; Alma et al. 2025). Genetic mutations in the MAPK/ERK pathway, such as BRAF, KRAS, and MAP2K1, have been reported in up to one-third of RDD cases, supporting a clonal neoplastic component in a subset of patients (Binhassan et al. 2025). In addition, immune dysregulation in RDD has been described but remains incompletely understood. Reported autoimmune conditions with potential association include autoimmune hemolytic anemia, immune thrombocytopenia, and antiphospholipid syndrome, hence the immune-disease-associated subtype (Vaiselbuh et al. 2014; Lopetegui-Lia et al. 2019).

The clinical course is typically indolent, and many patients require no systemic therapy. Consensus recommendations suggest that uncomplicated, asymptomatic disease can be conservatively managed, while symptomatic, refractory, or organ-threatening disease warrants tailored intervention with options such as surgical resection for focal lesions, corticosteroids, or other systemic therapies, including chemotherapy and targeted agents, depending on severity and response (Abla et al. 2018).

Hereby, we report an unusual presentation of a pediatric patient with biopsy-proven RDD associated with profound lymphopenia and significant immune dysregulation. We also review the reported cases in the literature presenting with lymphopenia in the context of RDD and discuss diagnostic considerations and implications for precision management.

## Case presentation

An otherwise healthy 3-year-old Caucasian boy was reviewed at a two-week wait clinic presenting with a one-month history of progressive bilateral neck swellings and intermittent dysphonia. Symptoms developed following a transient viral upper respiratory illness. There was no history of fever, weight loss, night sweats, fatigue, or other lumps or swellings. His past medical history was unremarkable, apart from a self-limiting episode of chickenpox during infancy. Family history was unremarkable apart from reports of early deaths among male relatives.

**Fig. 1:**
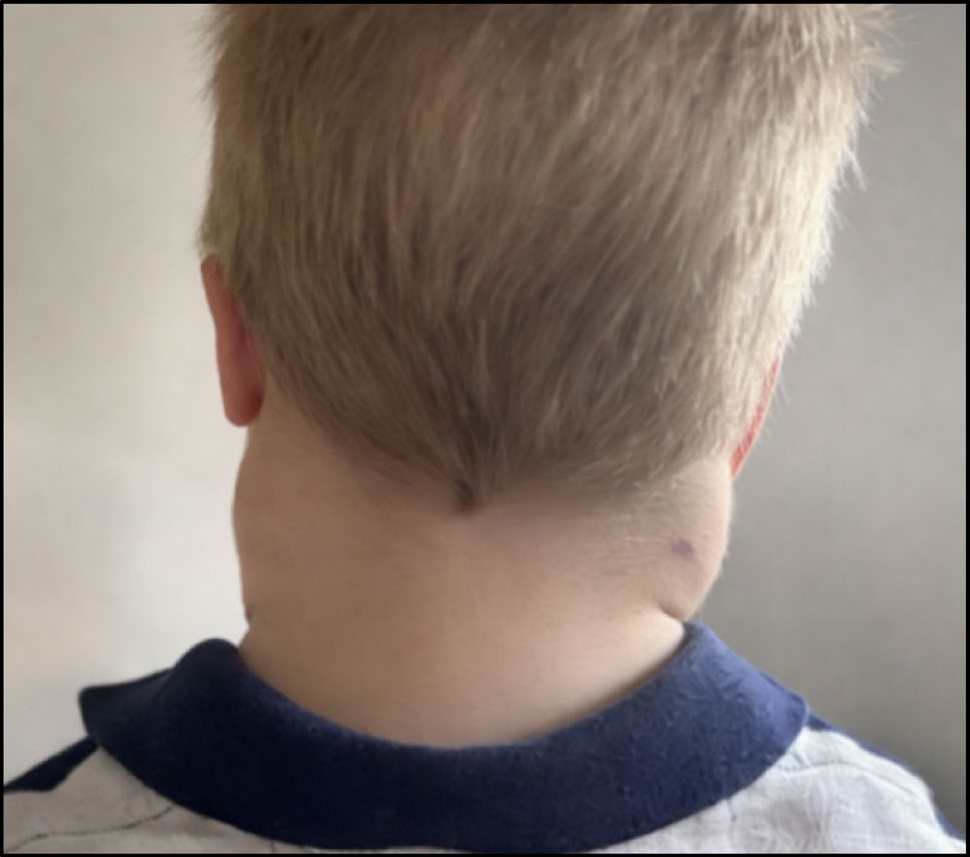
Clinical photograph demonstrating bilateral cervical lymphadenopathy, resulting in diffuse neck swelling and asymmetry

On physical examination, the patient was clinically well. He had extensive painless bilateral cervical and submandibular lymphadenopathy (Fig. 1). There was no evidence of skin lesions, hepatosplenomegaly, or other systemic abnormalities.

Initial routine blood tests were within normal limits except for profound lymphopenia. Serological testing for toxoplasmosis, cytomegalovirus, Epstein–Barr virus, mumps, and HIV was negative.

Immunophenotyping revealed remarkably low levels of CD3^+^, CD4^+^, and CD8^+^ T cells as well as CD19^+^ B cells with low borderline levels of CD56^+^/CD16^+^ NK cells. Double-negative T cells (CD3^+^/CD4^−^/CD8^−^) accounted for approximately 27% of T-cells and were predominantly TCRγδ^+^. In this case, the expansion of γδ T-cells was identified as a relative increase in proportion (percentage) within the T-cell compartment rather than a clear absolute expansion. Given the overall profound T-cell lymphopenia, the elevated percentage of double-negative TCRγδ^+^ cells likely reflects a relative predominance due to depletion of conventional αβ T cells. The B-cell subset analysis was performed using standard flow cytometric definitions: switched memory B cells (CD19^+^CD27^+^IgD^−^), marginal zone B cells (CD19^+^CD27^+^IgD^+^), naïve B cells (CD19^+^CD27^−^IgD^+^), and transitional B cells (CD19^+^CD38 > high> CD24> high>). The B-cell subset analysis demonstrated reduced switched memory B cells, markedly reduced marginal zone B cells, elevated naïve B cells, and markedly increased transitional B cells. These findings suggested immune dysregulation rather than classical autoimmune lymphoproliferative syndrome (Table 1).

**Table 1:** Immunophenotyping results in the present case demonstrating pan–T-cell lymphopenia with markedly reduced CD4^+^ T cells and borderline low CD4/CD8 ratio, reduced CD19^+^ B cells with decreased switched memory and marginal zone subsets, expansion of naïve and transitional B cells, and a markedly elevated proportion of double-negative T cells (CD3^+^/CD4^−^/CD8^−^)

Parameter	Patient Value	Age-Adjusted Reference Range	Interpretation
CD3^+^ T cells	0.34 × 10^9^ L	1.0–3.5 × 10^9^ L	Low
CD4^+^ T cells	0.13 × 10^9^ L	0.5–2.0 × 10^9^ L	Markedly low
CD8^+^ T cells	0.13 × 10^9^ L	0.3–1.2 × 10^9^ L	Low
CD4/CD8 ratio	~ 0.95	1.0–3.0	Borderline low
CD19^+^ B cells	0.10 × 10^9^ L	0.5–1.5 × 10^9^ L	Low
CD16^+^ CD56^+^ NK cells	0.12 × 10^9^ L	0.1–0.8 × 10^9^ L	Borderline
Double-negative T cells (CD3^+^/CD4^−^/CD8^−^)	27% of CD3^+^ cells	< 2%	Markedly elevated
Switched memory B cells	Reduced	Age-matched normal	Decreased
Marginal zone B cells	Markedly reduced	Age-matched normal	Decreased
Naïve B cells	Increased proportion	Age-matched normal	Expanded
Transitional B cells	Significantly increased	< 10% of B cells	Markedly expanded
Serum immunoglobulins	Within normal range	Age-matched normal	No hypergammaglobulinemia

Serum immunoglobulin levels were within the age-adjusted normal range. Summary of the reported RDD cases with cytopenia

**Fig. 2:**
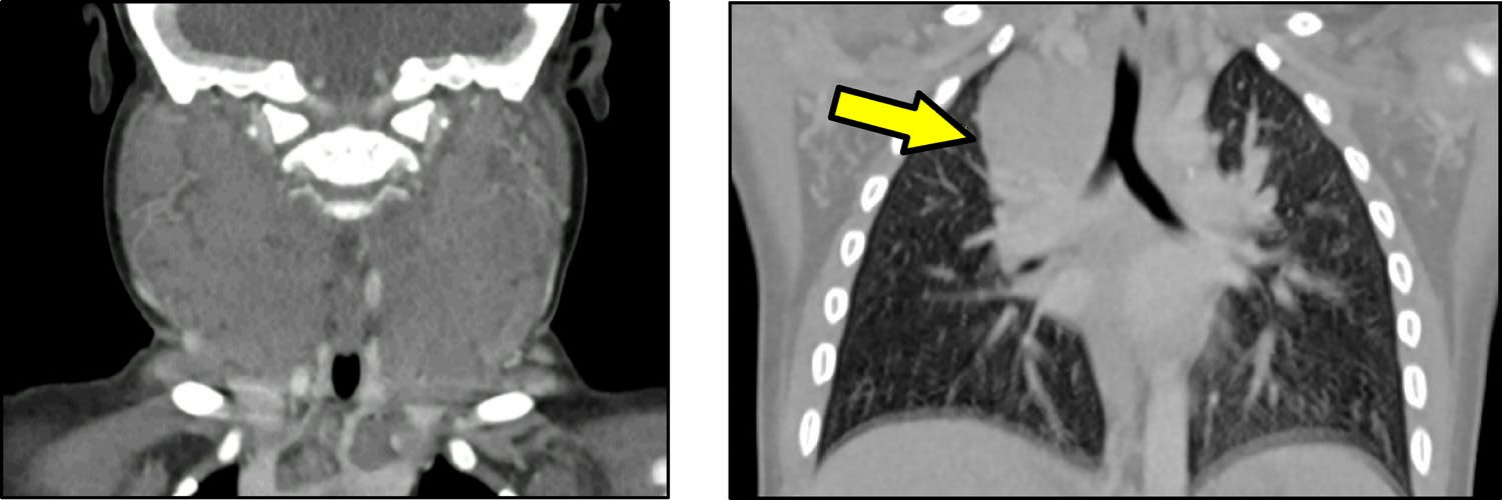
Contrast-enhanced CT images. The left image represents a coronal CT image of the neck demonstrating extensive bilateral cervical lymphadenopathy. The right image demonstrates a coronal CT image of the chest showing right mediastinal lymphadenopathy (yellow arrow) Immunophenotyping parameters of the present case

Computed tomography (CT) of the neck and thorax revealed extensive bilateral cervical lymphadenopathy and right superior mediastinal lymphadenopathy (Fig. 2). Abdominal and pelvic ultrasonography was normal.

An excisional biopsy of a right cervical lymph node demonstrated characteristic histopathological features of RDD, including sinus expansion by large histiocytes exhibiting emperipolesis (S100^+^/CD68^+^/CD1a^−^ histiocytes). Representative histopathological images were not available for inclusion; however, the diagnosis was established based on the histopathological report along with classical morphologic and immunophenotypic findings in conjunction with expert panel review.

Bone marrow testing demonstrated normal trilineage hematopoiesis, with no evidence of histiocytic infiltration, dysplasia, or malignancy. Additional flow cytometry or PCR-based studies were not performed, as there were no clinical, hematologic, or morphologic features suggestive of an underlying lymphoproliferative disorder or marrow pathology. Furthermore, the patient’s clinical stability and subsequent spontaneous resolution of lymphadenopathy without therapy supported a non-neoplastic process.

Following multidisciplinary review and assessment by the UK National Histiocytosis Advisory Panel, the diagnosis was confirmed, and further immunologic and genetic evaluation was recommended. Molecular testing included analysis of key RDD-associated MAPK pathway genes (*BRAF*,* KRAS*, and *MAP2K1*), with no pathogenic variants identified. In addition, targeted evaluation for autoimmune lymphoproliferative syndrome (ALPS)–related features was undertaken and did not reveal any abnormalities. Comprehensive autoantibody screening, including antinuclear antibodies and other relevant serologic markers, was negative, supporting the absence of an underlying autoimmune process.

Given the patient’s stable clinical condition and absence of extranodal disease or organ dysfunction, a conservative management approach was decided. The patient was therefore commenced on prophylactic co-trimoxazole due to significant lymphopenia and advised to avoid live vaccines. Although mTOR inhibitors such as sirolimus have been reported to be beneficial in bulky or progressive RDD, treatment was deferred because of clinical stability. Re-biopsy and further genetic analysis were planned in the event of disease progression or relapse. Regular follow-up every four months over 12 months with pediatric oncology and immunology services was arranged. During follow-up, the patient remained clinically stable with persistent but non-progressive lymphadenopathy and no evidence of disease progression. Notably, at the most recent follow-up, the patient’s lymphadenopathy has completely resolved, while lymphopenia persists but has shown partial improvement, highlighting the effective conservative management approach.

## Discussion

We report a rare presentation of a pediatric case of RDD and conduct a review of the literature, summarized in Table 2, demonstrating that only a small number (8 cases) of well-characterized cases of RDD with lymphopenia or pancytopenia have been reported, underscoring the rarity of this presentation.

**Table 2:** Summary of previously reported cases of RDD associated with isolated lymphopenia or pancytopenia, detailing demographic characteristics, clinical presentation, extranodal involvement, viral associations, hematologic abnormalities, immunologic dysregulation, genetic findings, bone marrow infiltration, treatment modalities, and clinical outcomes, alongside the features of the present case

Study	Age/Sex	Clinical presentation	Extranodal disease	Viral infection	Hematologic Abnormality	Immunologic dysregulation	Genetic defect	BM infiltration	Treatment	Outcome
Petschner et al. (2001)	59/F	Fatigue and generalized lymphadenopathy	Pleura, pericardium, retroperitoneal, spleen, and BM	No	Pancytopenia	ANA+, antiphospholipid IgM+, hypocomplementemia	No	Yes	Steroids, cyclophosphamide, rituximab, IVIG, splenectomy	Sustained remission
Moreno et al. (2004)	9/M	Cervical lymphadenopathy	Oral cavity	No	Severe lymphopenia (↓CD3+, ↓CD4+, ↓CD8+)	hypergammaglobulinemia	No	No	Steroids, 6-mercaptopurine, methotrexate	Recurrent disease
Pérez et al. (2008)	45/F	Fever and cervical lymphadenopathy	No	No	Total T lymphopenia (CD4 + depletion)	Polyclonal hypergammaglobulinemia	No	No	Immunomodulatory therapy	Clinical remission and improved lymphopenia
Kaffenberger et al. (2012)	60/F	Fever and widespread lymphadenopathy	Cutaneous and pulmonary	No	Pancytopenia and ↑ESR	No	Trisomy 8	Yes (MDS)	Supportive treatment	N/A
Cooper et al. (2016)	1.7/M	Fatigue, fever, and generalized lymphadenopathy	No	No	Pancytopenia	Positive Coombs test, lupus anticoagulant, anti-platelet antibodies	No	No	Steroids, vinblastine, rituximab, sirolimus	Sustained remission
Kapoor et al. (2018)	58/M	Fatigue, weight loss, and generalized lymphadenopathy	CNS	No	Pancytopenia and ↑ESR	polyclonal hypergammaglobulinemia	No	No	6-mercaptopurine and Prednisolone	Remission (6–8 years) before CNS manifestation
Gogia et al. (2022)	55/F	Cervical, axillary, and inguinal lymphadenopathy	Cutaneous	Yes (COVID-19, CMV IgM + and IgG+, EBV IgG+)	Lymphopenia, anemia, and ↑ESR	ANA + and hypocomplementemia	No	No	Methylprednisolone	Clinical remission and improved lymphopenia and anemia
Proskuriakova et al. (2024)	Late 50s/M	Fatigue and cervical and inguinal lymphadenopathy	Cutaneous, pulmonary, spleen	No	Normal (post-treatment severe cytopenia)	No	KRAS (G13C), POLE, NDE1, EZH2 mutations	No	Antimicrobials, steroids, rituximab, lenalidomide, sirolimus	Radiologic remission and improved cytopenia
Present case	3/M	Cervical lymphadenopathy and dysphonia	No	Yes (recent viral URTI)	Severe lymphopenia	Combined T- and B-cell lymphopenia with expansion of TCRγδ⁺ double-negative T cells and disrupted B-cell maturation	No	No	Observation and prophylactic antimicrobials	Stable disease

M, male; F, female; BM, bone marrow; COVID-19, coronavirus disease 19; CMV, cytomegalovirus; EBV, Epstein-Barr virus; URTI, upper respiratory tract infection; ESR, erythrocyte sedimentation rate; TCR. T-cell receptor; ANA, antinuclear antibody; MDS, myelodysplastic disease; IVIG, intravenous immunoglobulin; N/A, not available

RDD patients typically present with painless cervical lymphadenopathy associated with leukocytosis and, in some cases, autoimmune features (Abla et al. 2018). This case demonstrates an unusual association of RDD with severe lymphopenia and marked immune dysregulation. Although immune dysregulation and autoimmune cytopenias have been described in RDD, true lymphopenia or pancytopenia remains uncommon in this context (Moreno et al. 2004; Cooper et al. 2016).

The pathophysiological link between RDD and lymphopenia is poorly understood. Possible mechanisms may include immune-mediated destruction, bone marrow infiltration/suppression, or cytokine-mediated dysregulation (Kaffenberger et al. 2012; Cooper et al. 2016; Gogia et al. 2022). The predominance of the double-negative TCRγδ⁺ cells, rather than TCRαβ⁺ cells, in the current case distinguished it from ALPS (Matson and Yang 2020).

Hematologic abnormalities in RDD can broadly be categorized into three phenotypic patterns: *i*) normal leukocyte count or leukocytosis (the most common picture), *ii*) isolated lymphopenia, or *iii*) multilineage cytopenias or pancytopenia.

True T-cell lymphopenia has been reported in only a few cases. Moreno et al. (2004) and Cooper et al. (2016) described a pediatric patient with severe lymphopenia (depleted CD3⁺, CD4⁺, and CD8⁺ cells) associated with hypergammaglobulinemia and a relapsing disease despite the use of immunosuppressants. Pérez et al. (2008) reported total T-cell lymphopenia with depletion of CD4 + cells and polyclonal hypergammaglobulinemia in an adult patient who had clinical remission of symptoms following immunomodulation. In both cases, there was no bone marrow infiltration, suggesting a peripheral or immune-mediated mechanism rather than primary bone marrow failure. Both reports indicate that isolated lymphopenia can occur in the context of RDD; however, neither included a detailed characterization of the broader lymphocyte subset distribution.

On the other hand, multiple cases described pancytopenia in the context of RDD. Petschner et al. (2001) reported an adult RDD patient with pancytopenia in association with anti-phospholipid syndrome and immune dysregulation. Cooper et al. (2016) described recurrent autoimmune hemolytic anemia and thrombocytopenia in a pediatric RDD patient, ultimately achieving sustained remission of the disease with sirolimus. Gogia et al. (2022) presented a case of RDD associated with cytopenia and a deranged immune system following COVID-19. In addition, Kapoor et al. (2018) reported a case that progressed into neurological manifestation with pancytopenia associated with polyclonal hypergammaglobulinemia as the sole identified immunologic abnormality. These findings support the concept that RDD may coexist with or trigger systemic immune dysregulation. Similarly, autoimmune serologic abnormalities and immune-mediated cytopenias have been observed in RDD cases. Notably, all these cases with immune-mediated presentations required systemic immunosuppressive treatment.

Interestingly, two of the reported cases described cytopenia in RDD without a deranged immune system. Kaffenberger et al. (2012) presented a case with pancytopenia in a patient with trisomy 8 and a concurrent myelodysplastic syndrome, whereas Proskuriakova et al. (2024) described a case with normal hematologic parameters, but cytopenia occurred only after the initiation of immunosuppressive treatment. These two cases suggest that cytopenias with no underlying immune dysfunction may occur in RDD due to a coexisting hematologic/bone marrow pathology or as a side effect of immunosuppressants rather than direct RDD-related immune dysregulation.

Our patient differs significantly from previously reported cases. This current case demonstrated a profound combined T- and B-cell lymphopenia without pancytopenia, without autoimmune serologic markers, and without bone marrow suppression or infiltration. Evidence shows three potential mechanisms that may account for cytopenias in RDD: *i*) direct marrow infiltration or suppression (Petschner et al. 2001), *ii*) immune-mediated peripheral destruction (Cooper et al. 2016), and *iii*) systemic immune dysregulation affecting lymphocyte homeostasis (Moreno et al. 2004; Pérez et al. 2008). Our case most closely aligns with the third mechanism. The absence of marrow involvement and autoimmune markers, combined with severe lymphopenia, suggests a broader adaptive immune disruption.

In addition, this patient exhibited marked expansion of double-negative T cells (CD3⁺/CD4⁻/CD8⁻), predominantly of the TCRγδ^+^ phenotype. This immunologic profile has not been clearly described in any of the previous reports. Unlike earlier lymphopenic cases, our patient did not display hypergammaglobulinemia, and detailed B-cell subset analysis revealed increased transitional B cells with reduced switched memory and marginal zone compartments, suggesting disruption of peripheral lymphocyte maturation rather than isolated depletion (Morbach et al. 2010).

The presence of expanded double-negative T cells raises important diagnostic and management considerations, particularly with regard to ALPS. Although ALPS is characterized by TCRαβ⁺ double-negative T-cell expansion and defective FAS-mediated apoptosis, our patient lacked splenomegaly, autoimmune cytopenias, FAS mutation, and hypergammaglobulinemia. Furthermore, the predominance of TCRγδ⁺ double-negative T cells argues against classical ALPS. The distinction between RDD and ALPS is clinically important, as ALPS usually requires targeted immunomodulatory management and carries a higher risk for long-term lymphoma, warranting closer follow-up (Vignesh et al. 2017; Matson and Yang 2020).

Emerging molecular data further support the biological heterogeneity of RDD. Somatic mutations involving the MAP kinase pathway have been identified in subsets of patients, which tends to be an aggressive disease (Garces et al. 2017; Jafri et al. 2021). Refractory cytopenic RDD has also been associated with KRAS and related mutations. For instance, in the case described by Proskuriakova et al. (2024), Sirolimus produced radiologic remission in a patient with severe refractory cytopenias and documented KRAS mutation. Whether immune-dysregulated RDD represents a molecularly distinct subgroup remains unknown, as most lymphopenic pediatric cases have not undergone comprehensive genetic testing. Further molecular characterization in future studies will be necessary to clarify this possibility.

Notably, most reported lymphopenic or cytopenic cases required systemic and sometimes surgical (splenectomy) interventions due to progressive disease, autoimmune coexistence, or organ involvement. Systemic treatments may include corticosteroids, immunomodulators, rituximab, and mTOR inhibition. Sirolimus has demonstrated a particular efficacy in refractory immune-mediated cases (Cooper et al. 2016; Proskuriakova et al. 2024). In contrast, the current case has remained clinically stable with observation and antimicrobial prophylaxis alone. This outcome reinforces current recommendations that management in RDD should be individualized and guided by clinical picture, organ involvement, and disease progression rather than laboratory abnormalities solely.

Compared with reported cases in this literature, the current case represents one of the youngest individuals described with lymphopenic RDD and is the first clearly characterized case demonstrating combined T- and B-cell lymphopenia with γδ-predominant double-negative T-cell expansion in the absence of autoimmunity or marrow infiltration. These findings expand the recognized immunologic spectrum of RDD and suggest that lymphopenic RDD presentations may constitute a biologically heterogeneous subgroup distinct from marrow-infiltrative or autoimmune-associated phenotypes. In conclusion, cytopenia in RDD is uncommon and biologically diverse. Isolated severe lymphopenia without bone marrow involvement is extremely rare and remains poorly understood. Comprehensive immunophenotyping is essential in pediatric patients presenting with lymphadenopathy and lymphopenia to distinguish RDD from primary immunodeficiency syndromes and lymphoproliferative disorders. Future studies integrating detailed immune profiling with genomic sequencing may clarify whether immune-dysregulated RDD represents a discrete molecular subtype with implications for targeted therapy and precision medicine approaches.

**Statements and declarations**.

## Data Availability

The datasets generated during and/or analysed during the current study are available from the corresponding author on reasonable request.
